# Cross-sectional study on urinary metal concentrations in young adult residents of Emirate of Sharjah, United Arab Emirates

**DOI:** 10.1371/journal.pone.0312964

**Published:** 2024-11-05

**Authors:** Asmaa Masarani, Raghad Khaled, Bdour Hussein, Huda Alhammadi, Salma Al-Ali, Yahya Kinbaz, Shima A. Mohammad Zadeh, Tamer Shousha, Mouath Mousa, Mai M. Hassanein, Mohammad Semreen, Lucy Semerjian, Khaled Abass

**Affiliations:** 1 Environmental Health Sciences, College of Health Sciences, University of Sharjah, Sharjah, United Arab Emirates (UAE); 2 Department of Physiotherapy, College of Health Sciences, University of Sharjah, Sharjah, UAE; 3 Research Institute of Sciences and Engineering, University of Sharjah, Sharjah, UAE; 4 Department of Medicinal Chemistry, College of Pharmacy, University of Sharjah, Sharjah, UAE; 5 Sharjah Institute for Medical Research, University of Sharjah, Sharjah, UAE; 6 Research Unit of Biomedicine and Internal Medicine, University of Oulu, Oulu, Finland; University of Alcala Faculty of Medicine and Health Sciences: Universidad de Alcala Facultad de Medicina y Ciencias de la Salud, SPAIN

## Abstract

**Background:**

Human biomonitoring is crucial for regulatory toxicology, yet data on biomarker concentrations in the UAE are lacking. This study addresses this gap by analyzing urinary concentrations of 16 metals in UAE young adults, assessing correlations with personal characteristics, dietary patterns, and lifestyle habits.

**Methods:**

A cross-sectional pilot study was conducted among 144 randomly selected young adults (71 males and 73 females) from Sharjah, UAE, between January and March 2023. Participants provided urine samples, which were analyzed for 16 heavy metals using ICP-OES, and completed detailed questionnaires covering sociodemographic factors, lifestyle, and dietary habits. Descriptive statistics were used to summarize participant characteristics, and linear regression analysis was applied to explore associations between metal concentrations and factors such as gender, dietary habits, and exposure to environmental risks. Non-parametric tests, including the Mann-Whitney test, were used to assess differences by gender. Statistical significance was set at p < 0.05. Ethical approval was obtained, and informed consent was secured before participation

**Results:**

Significant findings include dietary influences on metal exposure, with rice consumption linked to higher metal concentrations. Sex differences were significant, with females showing elevated levels of arsenic, lead, and cadmium. Lifestyle factors, such as smoking and incense use, were correlated with increased barium and boron levels.

**Conclusion:**

This study highlights the significant role of dietary habits, especially the consumption of rice, in metal exposure among young adults in Sharjah. The findings highlight the urgent need for comprehensive human biomonitoring to understand environmental exposures and reform public health policies. The gender-specific differences in metal distribution suggest the necessity for targeted public health strategies. The study, however, is limited by its cross-sectional nature and the focus on a specific geographic area, warranting further research for broader generalizability. Future investigations, particularly on the impact of incense exposure on metal levels, are essential for developing comprehensive health interventions and preventive strategies in the UAE.

## 1. Introduction

Humans are increasingly exposed intentionally or unintentionally to a wide variety of hazardous chemicals and their mixtures, which are found in air, soil, water, and food [1]. Pesticides, organochlorines, toxic metals, and other chemical elements are some examples of environmental pollutants that have negative effects on human health [2]. Prolonged human exposure to such chemicals, even at low doses, was found to be linked to chronic diseases and cancer [3–5]. Heavy metals are found naturally in earth’s crust. Human exposure to heavy metals has significantly increased over the last 50 years, due to the exponential growth in the use of various products and industries [6]. These metals can enter the body through a variety of routes, including dermal or inhalational routes, or through heavy metal ingestion from contaminated food and/or drinking water [7]. Arsenic (As), cadmium (Cd), and lead (Pb) are well-known toxic metals/metalloids that have been linked to a number of human diseases as per strong experimental and epidemiological evidence [8].

Other metals, such as aluminum (Al), is linked to neurological disorders like Alzheimer’s disease [9]. Barium (Ba) is associated with cardiovascular diseases and muscle weakness [10]. Boron (B) can impact cognitive function [11]. Chromium (Cr) and strontium (Sr) are linked to kidney damage [12]. Cobalt (Co) is known to cause cardiomyopathy and thyroid problems [13]. Copper (Cu) is associated with Wilson’s disease and neurological symptoms [14]. Excess iron (Fe) can cause liver disease, while magnesium (Mg) deficiency is linked to cardiovascular problems and muscle spasms [15]. Manganese (Mn) is associated with neurological disorders [16]. Nickel (Ni) can cause lung and nasal cancers [17]. Silver (Ag) is linked to respiratory issues, neurological disorders, and kidney damage [18]. Thallium (Tl) is known to cause neurological and gastrointestinal symptoms, cardiovascular and kidney diseases, adverse birth outcomes, and impaired male reproductive health [19].

Studies on both human and experimental animals have also shown that exposure to these metals alters cell structure and substitutes cofactors for enzymatic activities; as a result, these metals are linked to carcinogenicity since they are chemically reactive and challenging to remove from the body [4, 20].

Blood, as well as invasive and non-invasive human tissues, have all been found to contain both essential and non-essential metals [21]. The internal dose of a chemical resulting from aggregate exposures from all possible exposure routes is measured by human biomonitoring (HBM) [20]. Therefore, up-to-date and high-quality scientific human biomonitoring studies are becoming increasingly important in modern regulatory toxicology and have been utilized more frequently as a tool for quantifying population exposure to chemicals as well as for investigating associations between biomarkers of these chemicals and human health outcomes, therefore, assisting in risk assessment, risk management, and public health decisions [22].

Rising temperatures and indoor air pollutants, including particulate matter (PM2.5 and PM10), NO_2_, SO_2_ and VOCs, have been identified as the primary causes of many health problems in the Gulf Cooperation Council (GCC) countries, which includes the United Arab Emirates (UAE), Bahrain, Kuwait, Oman, Qatar, and Kingdom of Saudi Arabia [23, 24]. The reason is that these countries have experienced rapid industrialization and urbanization which resulted in a rise in environmental contaminants [25]. Siddiqi et al. showed that the concentration of selected heavy metals in GCC ground waters exceeded allowable international standards [26]. Limited biomonitoring studies have been carried out in GCC countries to monitor environmental pollution using various animals, such as the laughing dove and *Tridacna maxima* [27, 28]. studies utilizing human biomonitoring have primarily focused on occupational exposure [29] or vulnerable population subgroups, such as pregnant and lactating mothers [30] and children [31].The general population was the subject of only a small number of studies [32–34]. However, the comparability and quality control of current HBM studies in GCC countries remain limited by the different study populations and chemicals.

Although human biomonitoring is widely recognized as an invaluable tool for risk assessment and policy development, the GCC region currently lacks comprehensive national HBM initiatives targeting the general population [35]. Within the United Arab Emirates (UAE), only a single study has examined selected trace element levels in the plasma and breast milk of well-nourished women. This study involved 209 mothers who were breastfeeding children aged 4 to 80 weeks [36]. In response to these existing research gaps and concerns, this investigation seeks to provide new data regarding urinary biomarker concentrations in adult UAE residents. Additionally, the current study aims to explore potential correlations between these biomarker concentrations and various general characteristics, dietary patterns, and lifestyle habits among the study participants.

## 2. Materials and methods

### 2.1. The Ethics of the research

This research protocol was approved by the University of Sharjah Research Ethics Committee (REC-22-12-21-S). Written informed consent was obtained from each adult participant before participation in the study. Participants were fully informed about the study’s purpose, procedures, and benefits, and were assured that there were no risks involved. Confidentiality and anonymity were strictly maintained, with all personal data securely stored and accessible only to the research team.

### 2.2. Study design and participants

In this cross-sectional study, we recruited a cohort of 71 healthy male and 73 healthy female participants through random selection from Emirate of Sharjah, (25°21′27″N 55°23′27″E) UAE, between the months of January and March of 2023.A total of 144 urine samples were collected to achieve a statistical power of 0.95, a common benchmark in scientific studies. This power level was chosen to maximize the likelihood of detecting meaningful differences in metal/metalloid concentrations across the population. Including 144 samples allows for robust statistical analyses, facilitating the assessment of significant trends and associations. These analyses are essential for gaining insights into metal/metalloid biomonitoring among adults. Our data collection approach was comprehensive, involving a detailed questionnaire. The questionnaire was distributed using a stratified random sampling approach to ensure a representative sample of the adult population. Participants were randomly selected and approached at various community locations to maximize diversity and representativeness. Our field team provided each participant with a paper-based questionnaire in person, which facilitated immediate collection and allowed for any necessary clarifications. The survey included questions on sociodemographic details, lifestyle choices, and dietary habits, such as the consumption of various food types, use of household chemicals, and other behaviors related to environmental exposure. Participants were asked about topics ranging from their smoking habits and the frequency of rice consumption to their intake of fresh and flesh foods, use of insecticides and incense, dietary supplement usage, source of drinking water, home air quality assessment, and the utilization of facemasks. Participants were informed about the study’s significance and the importance of providing accurate information, with written informed consent obtained to ensure ethical compliance and response reliability.

### 2.3. Sample collection and preservation

Following the baseline questionnaire survey, urine samples were collected into 50 mL sterile, metal-free polypropylene containers, employing a clean catch method, and subsequently stored at -20°C until further analysis. Participants were instructed to provide a mid-stream urine sample as their first void of the day. Each urine sample ranged from 15 mL to 25 mL in volume and subsequent analysis was conducted within a timeframe of 4 weeks.

### 2.4. Sample preparation and processing

Prior to analysis, aliquots of urine samples were filtered through 25 mm cellulose acetate syringe membrane (45 μm pore size) into clean 15 mL conical plastic tubes to reduce interferences from particles.

### 2.5. Sample analysis

Heavy metals and metalloids were determined in the sample filtrates using ICP-OES (iCAP 7400, ICP-OES Duo, ThermoFisher Scientific, USA). Sixteen heavy metals were analyzed in the samples. Among them, As and B are considered metalloids, while Al, Ba, Cd, Cr, Co, Cu, Fe, Pb, Mg, Mn, Ni, Ag, Sr, and Tl are classified as metals. The specific wavelengths (nm) used for measuring the selected metals/metalloid of concerns were As– 189.042 {478} (Axial); Al ‐ 308.215 {109} (Axial); Ba ‐ 493.409 {68} (Axial); B ‐ 249.678 {135} (Axial); Cd ‐ 226.502 {449} (Axial); Cr ‐ 205.560 {464} (Axial); Co ‐ 228.616 {447} (Axial); Cu ‐ 324.754 {104} (Axial); Fe ‐ 238.204 {142} (Axial); Pb ‐ 216.999 {455} (Axial); Mg ‐ 279.079 {121} (Axial); Mn ‐ 257.610 {131} (Axial); Ni ‐ 216.556 {455} (Axial); Ag ‐ 328.068 {103} (Axial); Sr ‐ 421.552 {80} (Axial); and Tl ‐ 190.856 {477} (Axial).

Working standards used for instrument calibration were prepared in concentrations of 10, 25, 50, 100, 250, 500, and 750 ppb of the targeted metals. Calibration was performed using seven operational standards and achieved calibration curves exhibited coefficients of determination (R2) above 0.99. The attained limits of detection (LOD) for analytes under study were as follows: 13.21 μg/L As, 13.62 μg/L Al, 6.34 μg/L B, 0.093 μg/L Ba, 0.24 μg/L Cd, 0.46 μg/L Cr, 1.53 μg/L Co, 1.22 μg/L Cu, 1.52 μg/L Fe, 34.02 μg/L Pb, 6.81 μg/L Mg, 0.22 μg/L Mn, 3.18 μg/L Ni, 1.88 μg/L Ag, 0.017 μg/L Sr and 15.25 μg/L Tl. All recorded heavy metal concentrations were expressed as μg/L (ppb).

### 2.6. Quality control and quality assurance

Analytical-grade reagents were used, and ultrapure water was utilized for solution and standard preparations. All glassware, before being used, was soaked in diluted nitric acid for 24 hours and subsequently rinsed with deionized water. The calibration standard solutions were prepared through stepwise dilution of a stock solution, which was made from a certified multi-element ICP standard (1,000 mg/L in diluted nitric acid, Certipur Merck Chemicals). For quality control during ICP-OES analysis of heavy metals, blanks were run with each batch of samples to monitor contamination of used reagents and a standard was re-run for every 10 samples analyzed as calibration check. Blanks run was achieved using laboratory grade deionized water to reduce errors in the metallic determination to the barest minimum. Prepared samples were run in triplicates and recorded metal concentrations were expressed as mean ± SD in μg/L. Relative standard deviations among replicates were always less than 15%. All experimental procedures were conducted at a controlled room temperature of 24°C, in accordance with well-established laboratory protocols.

### 2.7. Statistical analysis

The selection of 144 urine samples for this cross-sectional pilot study was based on achieving a statistical power of 0.95, a standard in scientific research [37]. This level of power ensures a reasonable chance of detecting meaningful differences in metal concentrations within the population. Additionally, with 144 samples, robust statistical analyses could be conducted to assess the significance of observed trends and associations, providing valuable insights into the biomonitoring of metals in the adult population.

**Fig 1:**
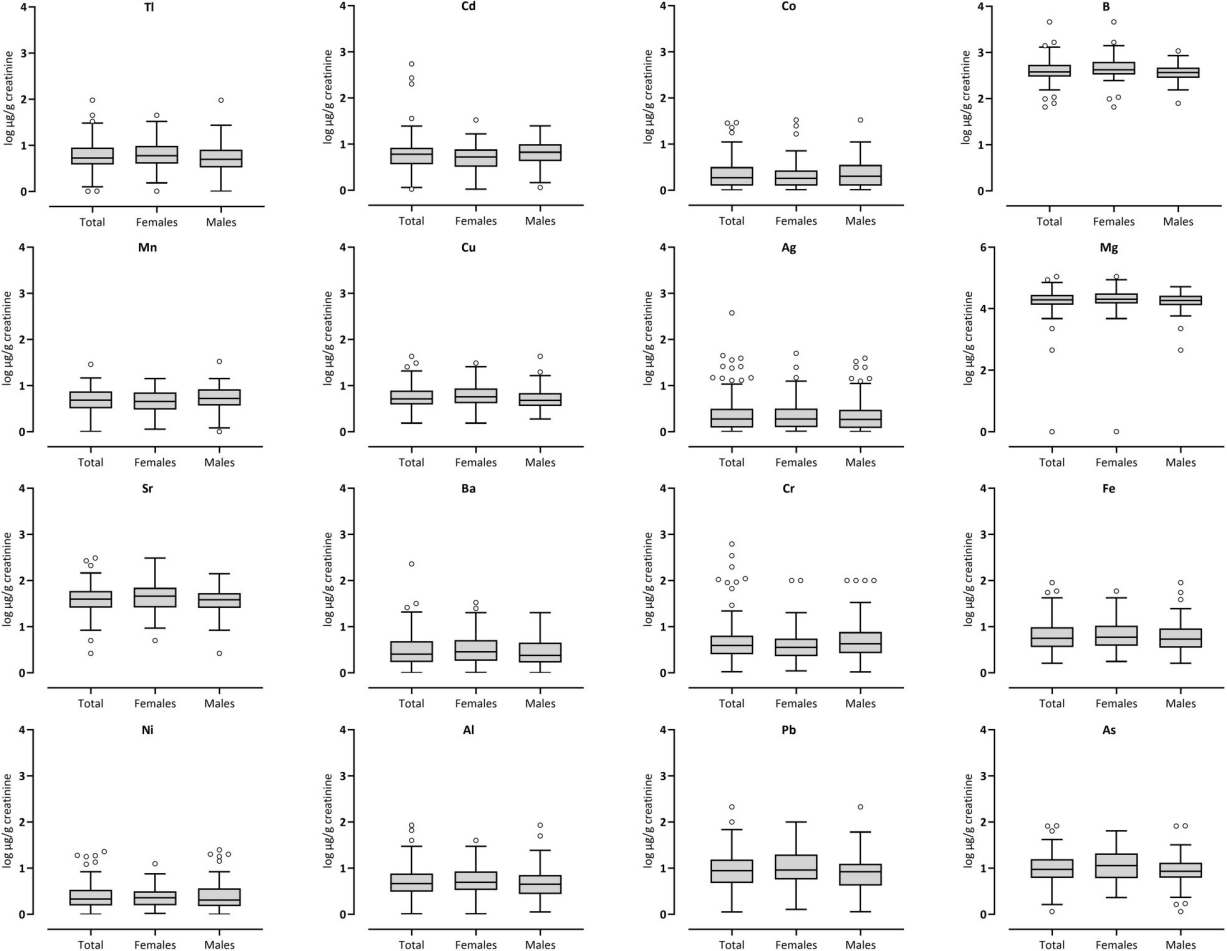
Box plots of metal concentrations (log μg/g creatinine) based on gender difference.

**Fig 2:**
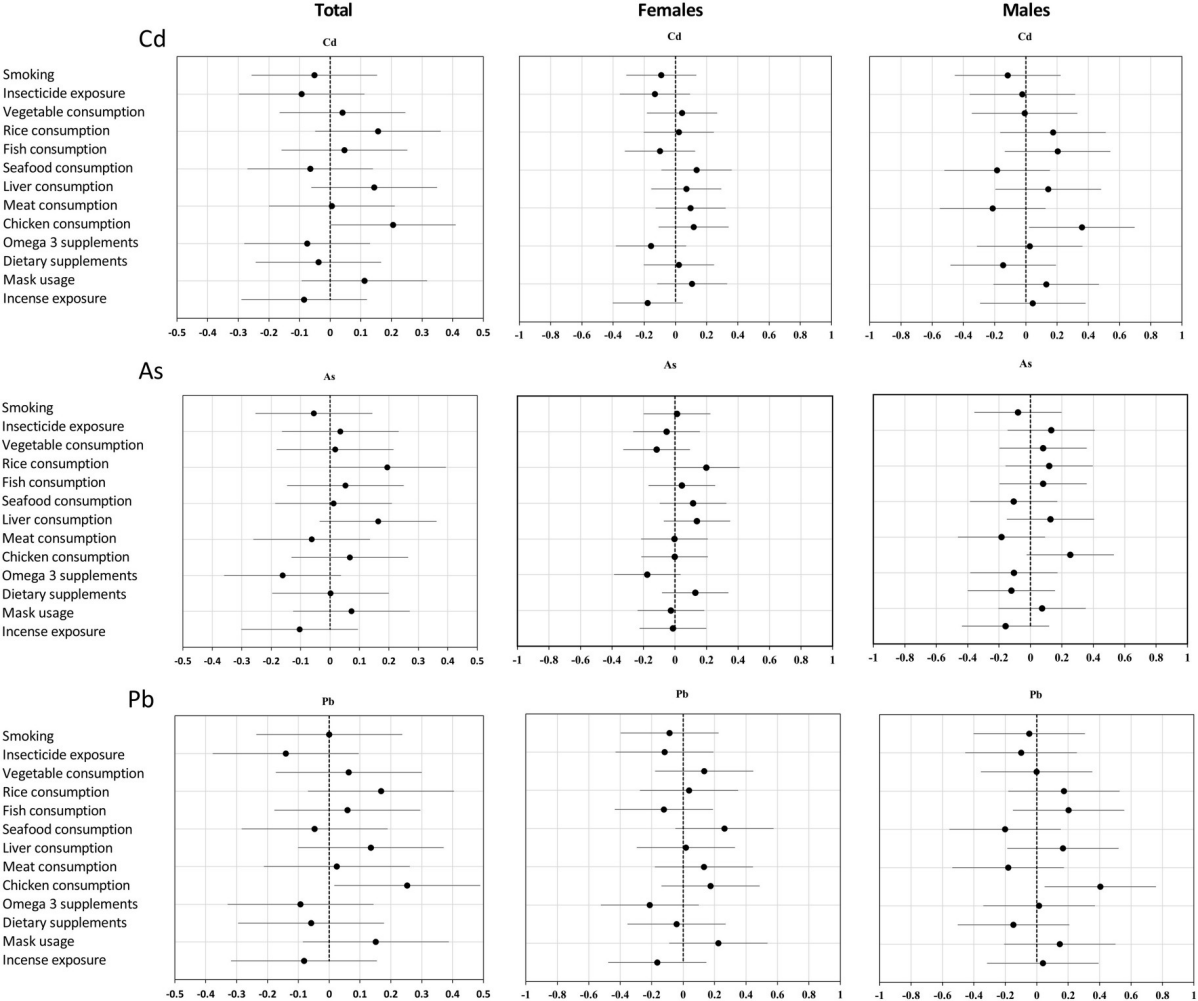
Standardized β-coefficients (dots) and 95% confidence interval (bars) of regression modelling of metal concentrations (Cd, Pb, and As) based on gender.

Following the descriptive analysis, the data from the collected samples were checked for normal distribution using the Kolmogorov-Smirnov and Shapiro-Wilk tests, revealing that all data were skewed. Descriptive analyses were carried out using percentiles, means, and standard deviations for continuous variables, as shown in Tables 1 and 2. Linear regression analysis was applied to explore the relationships between metal concentrations in urine and independent factors such as gender, dietary habits, exposure to facemasks, insecticides, smoking, and incense (S1 Table). Visual presentations of the data were provided as box plots in Figs 1, 2 and S1. The Mann-Whitney test was employed to investigate any gender-based differences (S2 Table and S2 Fig). Statistical significance was set at p < 0.05. All statistical analyses were performed using IBM SPSS Statistics version 28 (IBM Corp., Armonk, NY, USA).

**Table 1:** Characteristics of the study participants.

Variables	N	Mean (SD)	95% CI	Proportion (%)
Total	144			
Gender				
Female	73			50.69
Male	71			49.31
Age (years)				
Female	73	20.56 (1.80)	16.97–24.15	
Male	71	20.61 (1.66)	17.29–23.94	
All	144	20.58 (1.70)	17.18–23.98	
BMI (kg/m^2^)				
Female	73	24.28 (5.70)	22.95–25.61	
Male	71	24.66 (5.24)	23.42–25.90	
All	144	24.47 (5.46)	23.57–25.37	
Supplements intake				
Omega 3	27			18.75
Dietary	44			30.56
All	144			100.0
Face mask				
Yes	27			18.75
No	117			81.25
All	144			100.0
Incense exposure				
Yes	108			75.0
No	36			25.0
All	144			
Smoking status				
Smokers	21			14.58
2–4 days/week	9			6.25
5–7 days/ week	12			8.33
Secondhand smokers	37			25.69
Smoking prior sample	20			13.89
Never	123			85.42
All	144			100.0

SD; standard deviation, 95% CI; confidence interval, BMI; Body Mass Index

**Table 2:** Descriptive statistics of the metal concentration*.

Metals	Gender	N	Mean (SD)	P25	P50	P75	P90	P95	Range (Min-Max)
Tl	Female	73	8.60 (7.53)	4.01	5.96	9.84	17.12	25.83	0.98–44.86
	Male	71	7.50 (11.61)	3.32	5.02	8.03	10.83	20.52	0.31–95.33
	Total	144	8.05 (9.74)	3.84	5.32	8.97	15.12	21.04	0.31–95.33
Cd	Female		0.249 (0.22)	0.13	0.19	0.31	0.46	0.64	0.00–1.43
	Male		0.23 (0.37)	0.10	0.15	0.23	0.34	0.66	0.00–3.05
	Total		0.24 (0.30)	0.12	0.17	0.27	0.41	0.64	0.00–3.05
Co	Female		1.12 (1.12)	0.47	0.83	1.43	2.32	3.28	0.03–6.11
	Male		0.90 (1.25)	0.31	0.60	1.03	1.67	3.04	0.03–9.57
	Total		1.01 (1.19)	0.37	0.74	1.19	2.01	2.90	0.03–9.57
B	Female		564.10 (556.4)	331.4	421.7	629.0	870.1	1326	65.61–4591
	Male		395.20 (167.5)	281.5	368.7	470.4	608.2	703.4	79.27–1079
	Total		480.83 (420.5)	299.4	379.7	538.3	764.3	943.2	65.61–4591
Mn	Female		0.30 (0.26)	0.14	0.22	0.33	0.62	0.97	0.07–1.43
	Male		0.29 (0.46)	0.12	0.19	0.28	0.62	0.77	0.03–3.80
	Total		0.30 (0.37)	0.13	0.21	0.32	0.62	0.8	0.03–3.80
Cu	Female		7.63 (5.50)	4.17	5.72	8.73	15.70	20.06	0.65–30.73
	Male		6.24 (5.52)	3.61	4.76	6.89	10.38	14.52	0.53–43.14
	Total		6.94 (5.53)	3.90	5.17	7.83	13.23	18.69	0.53–43.14
Ag	Female		1.47 (1.91)	0.52	0.98	1.88	3.19	3.92	0.02–14.92
	Male		1.80 (4.66)	0.39	0.99	1.66	2.74	4.42	0.00–39.16
	Total		1.63 (3.54)	0.47	0.98	1.81	2.85	3.76	0.00–39.16
Mg*	Female		26.26 (18.4)	14.57	20.25	31.43	46.11	67.71	0.01–110.0
	Male		20.29 (10.1)	12.94	18.67	26.47	32.12	42.05	4.52–51.52
	Total		23.27 (15.1)	13.45	19.51	28.26	42.12	48.54	1.01–110.13
Sr	Female		56.53 (53.71)	25.71	45.67	67.89	109.44	165.00	0.01–307.38
	Male		42.98 (28.33)	25.35	38.4	53.19	83.40	111.55	2.65–139.88
	Total		49.85 (43.49)	25.68	39.09	58.47	94.12	132.71	0.01–307.38
Ba	Female		0.75 (1.02)	0.2	0.41	0.83	1.84	3.06	0.03–5.88
	Male		0.79 (1.30)	0.26	0.46	0.68	1.57	4.48	0.00–8.21
	Total		0.77 (1.16)	0.22	0.44	0.70	1.70	3.45	0.00–8.21
Cr	Female		0.41 (0.45)	0.18	0.28	0.45	0.72	1.48	0.00–2.73
	Male		0.40 (0.72)	0.13	0.24	0.38	0.66	1.36	0.00–5.80
	Total		0.40 (0.60)	0.16	0.26	0.4	0.65	1.34	0.00–5.80
Fe	Female		9.42 (10.14)	3.85	5.93	10.53	24.60	31.78	0.33–59.05
	Male		9.30 (12.98)	3.53	5.38	9.14	20.39	30.22	0.47–90.21
	Total		9.36 (11.59)	3.60	5.62	9.81	21.50	29.22	0.33–90.21
Ni	Female		1.82 (1.45)	0.65	1.63	2.67	3.61	4.75	0.08–7.57
	Male		1.58 (2.49)	0.37	0.90	1.92	3.28	5.84	0.04–17.86
	Total		1.70 (2.03)	0.50	1.21	2.30	3.36	4.62	0.04–17.86
Al	Female		6.99 (6.94)	3.22	4.93	8.51	13.58	23.07	0.29–40.06
	Male		6.19 (10.53)	2.33	4.08	6.81	9.43	18.32	0.02–0.02
	Total		6.59 (8.87)	2.76	4.51	7.63	12.97	18.79	0.02–85.12
Pb	Female		14.68 (16.02)	5.71	9.1	19.80	31.14	46.93	0.23–100.06
	Male		13.36 (26.25)	4.19	8.36	12.42	21.40	47.81	0.49–212.62
	Total		14.03 (21.61)	4.74	8.81	15.48	27.91	46.34	0.23–212.62
As	Female		14.06 (10.73)	6.06	11.39	20.88	27.50	31.52	0.30–64.30
	Male		11.95 (13.54)	6.19	8.58	13.11	22.21	29.36	0.61–82.55
	Total		13.02 (12.20)	6.16	9.39	15.69	25.69	30.36	0.30–82.55

* Expressed as μg/g creatinine; Number of participants: Female 73, Male 71, Total 144; The studied metals and metalloids were detected in all samples

## 3. Results

The main characteristics of the participants are shown in Table 1. The mean age was 20.58 ± 1.70 years with a BMI of 24.47 ± 5.46. 49.3% (71) of the studied population was male and 50.7% (73) was female. Masks were worn by 18.8% (27) of the participants, and incense was used by 75% (108). 85.4% (123) of the subjects had never smoked, while 8.3% (12) smoked on a regular basis (5–7 days per week). Almost half of the participants 49.4% (71) took dietary and omega 3 supplements.

The study determined median concentrations of various metals, normalized to creatinine levels, as shown in Table 2. The median concentrations were: Tl at 8.97 μg/g creatinine, Cd at 0.27 μg/g creatinine, Co at 0.74 μg/g creatinine, B at 379.72 μg/g creatinine, Mn at 0.21 μg/g creatinine, Cu at 5.17 μg/g creatinine, Ag at 0.98 μg/g creatinine, Mg at 19516.51 μg/g creatinine, Sr at 39.09 μg/g creatinine, Ba at 0.44 μg/g creatinine, Cr at 0.26 μg/g creatinine, Fe at 5.62 μg/g creatinine, Ni at 1.21 μg/g creatinine, Pb at 8.81 μg/g creatinine, and As at 9.39 μg/g creatinine. Additionally, (Fig 1) visually illustrates these metal concentrations, presented in both logarithmic scale and μg/g creatinine, highlighting differences between genders.

The outcomes of the linear regression analysis, exploring relationships between metal concentrations in urine and various factors such as gender, dietary habits (chicken, rice, seafood, vegetables, liver, and meat consumption), exposure to facemasks, insecticides, smoking, and incense, are presented in S1 Table. The findings highlight diet as the primary contributor to metal exposure in both genders. Notably, associations between metal concentrations (Tl, Cu, Co, Cd, Mn, Ag, Sr, Ba, Fe, Al, Pb, As, and Cr) and specific dietary components were observed (S1 Fig). Additionally, smoking exposure was associated with elevated concentrations of Ba (p < 0.05), and incense use was linked to increased levels of B (p < 0.05). Females showed higher concentrations of As, Pb, and Cd compared to males, indicating significant gender-based variations in metal concentrations. Noteworthy associations were found with seafood consumption for As, meat consumption for Pb, and incense exposure for Cd (Fig 2).

## 4. Discussion

### 4.1. Comparative analysis across GCC and global populations

The examination of geometric mean values elucidating metal concentrations in urine samples across various populations within the GCC countries has revealed intriguing findings, as summarized in Table 3. Notably, it was observed that approximately 42% of participants in the current study exceeded the geometric urine level of cadmium recommended by the Centers for Disease Control and Prevention (CDC). The CDC guideline for the general population (aged ≥6 years) establishes a threshold of 0.193 μg/g creatinine (equivalent to 0.185 μg/L) for cadmium [38].

**Table 3:** Comparison of geometric mean values of metals in urine samples from various populations in GCC countries: Current study vs. previously reported studies.

Element	Country	N	Population characteristics	Year of sampling	Population group (age)	Mean	Source
Cadmium (Cd)	UAE	144	Healthy female adults	2023	16–24	0.24 μg/g cr	Current study
	Qatar	239	farm workers who are currently working and residing in farms in Qatar	2012–2013	<20->50	1.09 μg/L	[41]
	Saudi Arabia	995	Healthy Saudi non-smokers and non-occupationally exposed women in Al-Riyadh city	2011–2013	17–48	0.96 μg/g cr	[40]
	Saudi Arabia	400	Healthy Volunteers	NA	20->70	0.54 μg/L	[39]
	Saudi Arabia	400	Deceased subjects	NA	20->70	1.56 μg/L	[39]
Cobalt (Co)	UAE	144	Healthy female adults	2023	16–24	1.01 μg/g cr	Current study
	Qatar	239	farm workers who are currently working and residing in farms in Qatar	2012–2013	<20->50	0.47 μg/L	[41]
Manganese (Mn)	UAE	144	Healthy female adults	2023	16–24	0.30 μg/g cr	Current study
	Qatar	239	farm workers who are currently working and residing in farms in Qatar	2012–2013	<20->50	2.15 μg/L	[41]
	Saudi Arabia	206	Healthy Saudi lactating mothers	2011–2013	19–45	3.06 μg/L	[42]
	Saudi Arabia	206	Healthy Saudi infants	2011–2013	2.9–12.39 months	3.68 μg/L	[42]
Copper (Cu)	UAE	144	Healthy female adults	2023	16–24	6.94 μg/g cr	Current study
	Qatar	239	farm workers who are currently working and residing in farms in Qatar	2012–2013	<20->50	15.0 μg/L	[41]
Silver (Ag)	UAE	144	Healthy female adults	2023	16–24	1.63 μg/g cr	Current study
	Saudi Arabia	400	Healthy Volunteers	NA	20->70	0.91 μg/L	[39]
	Saudi Arabia	400	Deceased subjects	NA	20->70	2.78 μg/L	[39]
Barium (Ba)	UAE	144	Healthy female adults	2023	16–24	0.77 μg/g cr	Current study
	Qatar	239	farm workers who are currently working and residing in farms in Qatar	2012–2013	<20->50	22.5 μg/L	[41]
Chromium (Cr)	UAE	144	Healthy female adults	2023	16–24	0.40 μg/g cr	Current study
	Qatar	239	farm workers who are currently working and residing in farms in Qatar	2012–2013	<20->50	0.55 μg/L	[41]
Iron (Fe)	UAE	144	Healthy female adults	2023	16–24	9.36 μg/g cr	Current study
	Qatar	239	farm workers who are currently working and residing in farms in Qatar	2012–2013	<20->50	34.1 μg/L	[41]
Nickel (Ni)	UAE	144	Healthy female adults	2023	16–24	1.70 μg/g cr	Current study
	Qatar	239	farm workers who are currently working and residing in farms in Qatar	2012–2013	<20->50	2.95 μg/L	[41]
Lead (Pb)	UAE	144	Healthy female adults	2023	16–24	14.03 μg/g cr	Current study
	Qatar	239	farm workers who are currently working and residing in farms in Qatar	2012–2013	<20->50	2.50 μg/L	[41]
	Saudi Arabia	206	Healthy Saudi lactating mothers	2011–2013	19–45	6.794 μg/L	[42]
	Saudi Arabia	206	Healthy Saudi infants	2011–2013	2.9–12.39 months	6.054 μg/L	[42]
	Saudi Arabia	995	Healthy Saudi non-smokers and non-occupationally exposed women in Al-Riyadh city	2011–2013	17–48	10.8 μg/g cr	[40]
	Saudi Arabia	400	Healthy Volunteers	NA	20->70	1.31 μg/L	[39]
	Saudi Arabia	400	Deceased subjects	NA	20->70	17.80 μg/L	[39]
Arsenic (As)	UAE	144	Healthy female adults	2023	16–24	13.02 μg/g cr	Current study
	Qatar	239	farm workers who are currently working and residing in farms in Qatar	2012–2013	<20->50	47.8 μg/L	[41]
	Saudi Arabia	400	Healthy Volunteers	NA	20->70	18.32 μg/L	[39]
	Saudi Arabia	400	Deceased subjects	NA	20->70	3.00 μg/L	[39]

Specifically, investigations in the GCC region, including studies conducted in Saudi Arabia by Issa et al. [39] and Al-Saleh [40], have reported elevated cadmium levels in urine. Issa et al. observed levels of 0.54 μg/L and 1.56 μg/L in healthy volunteers and deceased individuals, respectively. Al-Saleh focused on healthy Saudi non-smokers and non-occupationally exposed women in Al Riyadh city, reporting a cadmium level of 0.96 μg/g creatinine. Furthermore, a study in Qatar involving healthy male adult farm workers residing and working on farms documented a cadmium level of 1.09 μg/L [41]. Importantly, all these studies revealed cadmium levels in urine that exceeded the CDC-established cut-off point.

In our UAE-based investigation, both male and female subjects exhibited a mean Pb concentration of 14.03 μg/g cr, aligning with similar Pb studies conducted in Qatar and Saudi Arabia (Table 3). These studies encompassed farm workers in Qatar, healthy Saudi lactating mothers, infants [42], healthy Saudi non-smokers, non-occupationally exposed women in Al Riyadh city, as well as healthy volunteers and deceased subjects.

Meanwhile, the total concentration of the As element in the UAE stood at 13.02 μg/g cr. However, due to the lack of creatinine adjustments in human biomonitoring geometric mean values, direct comparisons with studies conducted in Qatar and Saudi Arabia are precluded. Unfortunately, a direct comparison of geometric mean values between our UAE-based study and these counterparts proves challenging due to the absence of creatinine-adjusted values in the datasets of these studies and the distinct characteristics of the study populations. These differences underscore the importance of standardizing methodologies and reporting formats in studies assessing heavy metal concentrations across diverse populations. Standardization facilitates meaningful inter-study comparisons and contributes to a comprehensive understanding of regional patterns of exposure.

In addition to comparing our findings with data published in the GCC countries, we also examined the concentrations of metals observed in various populations worldwide. A study conducted in China between 2017 and 2018, which included 807 individuals aged 3 to 79 years, reported higher median concentrations of As (25.2 μg/L Cr), Ni (2.69 μg/L Cr), Cr (1.99 μg/L Cr), Cd (0.45 μg/L Cr), and Tl (0.30 μg/L Cr) than those observed in our study. However, Pb (0.76 μg/L Cr) and Co (0.28 μg/L Cr) levels were lower. Interestingly, Mn was not detected in the Chinese study (Cheng et al., 2023).

In Iran, Soleimani et al. (2024) reported higher mean concentrations of Al (195.45 μg/g Cr), As (17.58 μg/g Cr), Cd (0.65 μg/g Cr), Cr (15.76 μg/g Cr), Pb (18.11 μg/g Cr), and Ni (17.78 μg/g Cr) in 490 participants aged above 25 years. These values exceeded those found in our study. Similarly, another study by Soleimani et al. (2024) among 100 urban residents in Iran revealed higher concentrations of Al (181.95 μg/L Cr), As (20.97 μg/L Cr), Cd (0.74 μg/L Cr), Cr (15.14 μg/L Cr), Ni (17.68 μg/L Cr), and Pb (17.89 μg/L Cr) compared to our results.

In the French population, the French Nutrition and Health Survey (ENNS) from 2006–2007 found a geometric mean total arsenic level of 3.34 μg/g Cr for adults aged 18–74 years, which was lower than the values observed in our study (Saoudi et al., 2012). Additionally, the Esteban study (Oleko et al., 2024) reported higher levels of Al (14.14 μg/g Cr), total As (27.74 μg/g Cr), Ba (4.16 μg/g Cr), Cd (0.57 μg/g Cr), Cu (12.09 μg/g Cr), and Ni (2.04 μg/g Cr) in adults, although the concentrations of Co (0.55 μg/g Cr), Mn (0.25 μg/g Cr), Pb (0.22 μg/g Cr), and Ti (0.257 μg/g Cr) were lower than those observed in our study.

Our findings reveal that the levels of several metals in the UAE population are generally lower than those reported in other regions, such as China and Iran, but comparable to or higher than those in France. Notable differences in metal concentrations across global populations emphasize the influence of regional environmental factors, dietary habits, and varying levels of industrial exposure. These findings highlight the importance of context-specific biomonitoring to better understand the environmental and public health implications of heavy metal exposure.

### 4.2. Metal distribution based on gender differences

The impact of heavy metal exposure appears to exhibit gender-specific variations, as suggested by current research. Analysis of average levels of specific elements and the establishment of reference ranges revealed notable distinctions between genders (S2 Table and S2 Fig). Statistically significant differences were observed, particularly in the case of Tl, B, Cd, Cu, and Ni content. Females exhibited significantly higher levels of these elements, with increases of 21% for both Tl and Cu, and 19%, 25%, and 24% for Cd, B, and Ni, respectively. The elevated Cd levels in females can be attributed to increased intestinal absorption when iron stores are depleted, a condition more common in women due to monthly menstruation. In our study, there were 5 females and 19 males who were obese (with BMI >30). Obesity as a cofactor in heavy metal levels in the study population requires a larger number of individuals and further study. Marketing targeting women, through jewelry, personal care products, and cosmetics, as well as exposure to incense, can also contribute to higher Cd levels [43]. Despite limited information on associations between heavy metals and sex hormones, understanding gender-specific sensitivity to heavy metals is crucial for effective public health measures. In our study, all participants were healthy and reported having no chronic diseases. Additionally, all female participants (age range 18–26 yrs.) were not menstruating during the sample collection period. However, establishing conclusive causal relationships is challenging due to the cross-sectional nature of existing studies, where metal exposures and hormone levels are measured at single time points. Further investigation with larger cohort studies is required [44–46].

### 4.3. Diet

The comprehensive examination of metal concentrations in urine and their associations with dietary factors in the study revealed numerous significant correlations (P value > 0.05), with rice exhibiting the most pronounced associations in both male and female urine samples. The identified metals include Tl, Cu, Co, Cd, Mn, Ag, Fe, Al, Pb, As, and Cr. Such observation highlights the substantial role of rice, a dietary staple in the Gulf area, in contributing to the intake of these metals among young adults in the UAE. Notably, the reported rice consumption of 77g per capita per day underscores its significance in the local diet [47]. Gender-specific associations further emerged, exemplified by significant correlations between rice consumption and Ni and Ba in male urine samples (P value <0.01). While the reasons behind these associations remain partially understood, potential sources such as metal content in rice cultivation soil or irrigation water/wastewater, or contamination during processing have been suggested [48]. Akoury et al. examined As levels in 129 rice samples in the UAE, revealing an average of 0.18 ± 0.09 mg/kg in dry rice, below the Codex Alimentarius Commission (CAC) limit of 0.3 mg/kg. However, 9% of samples exceeded this threshold, prompting consideration of factors like packaging season, origin, and harvesting timing that influence heavy metal levels in UAE rice [49]. This underscores the urgency for further research to validate these findings and assess associated health risks.

Beyond rice, the study identified noteworthy associations between the presence of arsenic in urine and seafood consumption among females. The broad category of seafood, including finfish, shellfish, and seaweed, emerges as a primary global source of arsenic exposure [50]. Although various biomonitoring studies have explored the link between seafood consumption and urinary arsenic levels, the precise reasons for these associations remain elusive. Potential sources, including arsenic in the water where seafood is harvested or contamination during processing, have been proposed [51]. Given the significance of seafood as a staple food source in Gulf countries, concerns regarding the bioaccumulation of heavy metals through the water, sediment, and food chain have surfaced. This is particularly critical as arsenic exposure has been linked to skin diseases, neurological disorders, cancers, diabetes mellitus, and cardiovascular diseases [52].

Livestock products, such as chicken, meat, and organ meats like liver, constitute integral components of the traditional diets in GCC nations, including the UAE. The study identified a significant relationship between liver consumption and urinary arsenic levels in Emirati young adults. This correlation aligns with broader global research indicating a link between dietary habits and arsenic exposure. The liver’s role in detoxification renders it susceptible to accumulating contaminants, including heavy metals. Studies from various regions have demonstrated instances where arsenic levels in liver samples exceeded established standards, emphasizing the impact of industrial activities. This necessitates further investigations to comprehensively understand the implications of dietary choices on heavy metal exposure [53–56].

### 4.4. Life characteristics

In this study, the relationship between dietary supplements and omega-3 consumption, face mask use, insecticide and incense exposure, and cigarette smoking was examined. No significant relationships were observed between face mask use or omega-3 consumption and any of the 16 metals studied. However, insecticide exposure was found to be significantly associated with Tl, Ni, Cu, Mn, Fe, Al, and Cr in females’ urine samples. While no reported mechanisms of interaction between heavy metals and pesticides exist, it is recognized that the co-presence of pesticides and heavy metals may influence each other’s toxicity, potentially enhancing or mitigating the resulting toxic effects and contributing to cumulative xenobiotic effects. Although the individual toxicity mechanisms of heavy metals and pesticides are well-documented, the synergistic toxicity mechanism of these xenobiotics remains unclear, warranting further focused studies in this area [57]. Similarly, incense exposure was significantly associated with Tl, Cu, Co, Cd, and Fe in females’ urine samples, and B in both males and females. The common practice of burning Arabian incense (Bakhour) in the Middle East and Arabian Gulf, particularly among females in the UAE, was highlighted. Prior research by Elsayed et al. revealed that Bakhour could sequester high amounts of metals such as Cd, Ni, Cu, Zn, Fe, and Pb from soils, posing potential health risks [58]. Cadmium, in particular, is a toxic metal associated with severe effects on the lungs, kidneys, and bones, as well as carcinogenic properties and respiratory issues [59]. Dietary supplements showed significant correlations with Ni in females’ urine samples and Sr in males’ urine samples. The presence of Ni, a non-essential and highly toxic element, in food supplements is noteworthy and should be considered as contamination [60]. Additionally, strontium, sometimes used as a dietary supplement in forms such as strontium citrate or strontium ranelate, has garnered attention for its potential benefits in supporting bone health. Research indicates that strontium may enhance bone mineral density and reduce fracture risks, particularly relevant for individuals dealing with osteoporosis or osteopenia [61].

### 4.5. Limitations and strengths

While this study provides valuable insights, it is essential to acknowledge its limitations. One notable constraint is the scarcity of comprehensive data available for comparison within the UAE and Gulf countries. The limited data, even in countries like Saudi Arabia, often lacks creatinine correction, posing challenges for accurate comparisons. Additionally, the experimental nature of our study, conducted with a pilot sample, implies a relatively small sample size, and future studies should prioritize expanding the participant pool to enhance generalizability and statistical robustness. On a positive note, the study capitalizes on specific age criteria by focusing exclusively on young adults. The inclusion of both genders, males, and females, adds depth to the analysis and interpretation of findings. Notably, a key strength lies in the meticulous synchronization of data collection, as samples were obtained from a consistent age group of adults, gathered during the early morning within a three-month timeframe. This approach ensures a more cohesive dataset, minimizing potential confounding variables and enhancing the internal validity of the study.

Our study’s limitations, including the limited sample size and the challenge of comparing our findings with those from other GCC countries due to methodological differences, suggest the need for standardized approaches in future HBM studies. Such standardization would enable more accurate and comprehensive assessments of heavy metal exposure across different populations.

## 5. Conclusion

Our findings reveal elevated concentrations of various heavy metals in the adult population of Sharjah, UAE, with dietary patterns, particularly rice and seafood consumption, emerging as significant contributors. The gender-specific variations in metal exposure, notably higher levels of certain metals in females, highlight the need for targeted public health interventions and policy considerations. The utilization of human biomonitoring in this study provides crucial insights into the internal exposure levels of heavy metals in the population. The observed associations between heavy metals and various lifestyle factors, such as dietary habits, incense use, and exposure to insecticides, emphasize the complex interplay of environmental and behavioral factors in determining heavy metal exposure. Furthermore, the significant correlations found between certain heavy metals and specific dietary elements underscore the urgent need for monitoring and regulating the presence of these metals in food products.

In conclusion, our study highlights the critical need for ongoing monitoring and public health initiatives to address heavy metal exposure in the UAE and the broader GCC region. It also calls for more extensive research to better understand the long-term health effects of chronic exposure to these metals. By improving our knowledge of these exposure pathways and their health impacts, we can better protect public health and inform policy decisions in the region.

## Supporting information

S1 Table Linear regression of the urine metal concentrations in adult UAE residents and different variables of exposure. (DOCX)

S2 Table Independent-samples Mann-Whitney U test summary. (DOCX)

S1 Fig Standardized β-coefficients (dots) and 95% confidence interval (bars) of regression modelling of metal concentrations based on gender. (TIF)

S2 Fig Independent-samples Mann-Whitney U test for gender differences. (JPG)
