# Supplementary material for: Cross-sectional study on urinary metal concentrations in young adult residents of Emirate of Sharjah, United Arab Emirates
Source: PLoS One. 2024 Nov 5;19(11):e0312964. doi: 10.1371/journal.pone.0312964 (PMC11537376; doi:10.1371/journal.pone.0312964)
Supplement: S1 Table — (DOCX) [file pone.0312964.s001.docx]

**Table S1: Linear regression of the urine metal concentrations in adult Sharjah residents and different variables of exposure.**

| **variables** | **N** | **Gender** | **Tl** | **Ni** | **Cu** | **B** | **Co** | **Cd** | **Mn** | **Ag** | **Mg** | **Sr** | **Ba** | **Fe** | **Al** | **Pb** | **As** | **Cr** |
| --- | --- | --- | --- | --- | --- | --- | --- | --- | --- | --- | --- | --- | --- | --- | --- | --- | --- | --- |
|  |  |  | P | P | P | P | P | P | P | P | P | P | P | P | P | P | P | P |
| **Chicken consumption**   - **0-1 time days/week** - **2-4 times days/week** - **5-7 times days/week** | **2**  **122**  **20** | **Female** | 0.461 | 0.391 | 0.308 | 0.214 | 0.125 | 0.412 | 0.189 | 0.279 | 0.394 | 0.406 | 0.217 | 0.458 | 0.361 | 0.325 | 0.435 | 0.486 |
|  |  | **Male** | **0.002** | **0.001** | **0.002** | 0.261 | **0.007** | **0.001** | **0.001** | **0.002** | 0.320 | 0.423 | 0.119 | **0.002** | **0.003** | **0.000** | 0.094 | **0.002** |
|  |  | **Total** | **0.011** | 0.006 | 0.080 | 0.235 | **0.006** | **0.006** | **0.002** | **0.011** | 0.293 | 0.352 | 0.359 | **0.016** | **0.011** | **0.001** | 0.151 | **0.011** |
| **Rice consumption**   - **0-1 time days/week** - **2-4 times days/week** - **5-7 times days/week** | **1**  **109**  **34** | **Female** | 0.364 | 0.053 | 0.135 | 0.467 | 0.499 | 0.406 | 0.386 | 0.407 | 0.346 | 0.466 | 0.274 | 0.247 | 0.335 | 0.491 | 0.088 | 0.419 |
|  |  | **Male** | **0.003** | **0.005** | **0.001** | 0.138 | **0.001** | **0.002** | **0.003** | **0.013** | 0.135 | 0.146 | **0.007** | **0.004** | **0.005** | **0.001** | 0.061 | **0.009** |
|  |  | **Total** | **0.006** | 0.079 | **0.001** | 0.342 | **0.009** | **0.012** | **0.007** | **0.029** | 0.150 | 0.293 | 0.068 | **0.006** | **0.008** | **0.004** | **0.016** | **0.018** |
| **Seafood consumption**   - **0-1 time days/week** - **2-4 times days/week** - **5-7 times days/week** | **57**  **87**  **0** | **Female** | 0.109 | 0.255 | 0.302 | 0.221 | 0.249 | 0.300 | 0.209 | 0.248 | 0.229 | 0.294 | 0.314 | 0.154 | 0.260 | 0.093 | **0.048** | 0.397 |
|  |  | **Male** | 0.148 | 0.343 | 0.278 | 0.310 | 0.148 | 0.248 | 0.247 | 0.114 | 0.493 | 0.099 | 0.205 | 0.356 | 0.195 | 0.210 | 0.390 | 0.098 |
|  |  | **Total** | 0.417 | 0.490 | 0.469 | 0.297 | 0.368 | 0.375 | 0.424 | 0.201 | 0.239 | 0.153 | 0.169 | 0.365 | 0.357 | 0.498 | 0.213 | 0.170 |
| **vegetables consumption**   - **0-1 time days/week** - **2-4 times days/week** - **5-7 times days/week** | **111**  **33**  **144** | **Female** | 0.408 | 0197 | 0.472 | 0.297 | 0.428 | 0.294 | 0.480 | 0.288 | 0.491 | 0.318 | 0.162 | 0.454 | 0.310 | 0.350 | 0.128 | 0.106 |
|  |  | **Male** | 0.412 | 0.267 | 0.482 | 0.264 | 0.453 | 0.477 | 0.418 | 0.309 | **0.043** | **0.008** | 0.097 | 0.291 | 0.474 | 0.492 | 0.221 | 0.487 |
|  |  | **Total** | 0.409 | 0.151 | 0.443 | 0.461 | 0.471 | 0.417 | 0.441 | 0.237 | 0.154 | **0.049** | 0.057 | 0.370 | 0.379 | 0.438 | 0.465 | 0.225 |
| **Liver consumption**   - **0-1 time days/week** - **2-4 times days/week** - **5-7 times days/week** | **71**  **73**  **0** | **Female** | 0.131 | 0.432 | 0.078 | 0.336 | 0.300 | 0.316 | 0.304 | 0.140 | 0.149 | **0.012** | 0.208 | 0.287 | 0.179 | 0.329 | 0.077 | 0.225 |
|  |  | **Male** | **0.089** | **0.015** | **0.041** | 0.319 | 0.202 | 0.093 | 0.085 | 0.181 | 0.349 | 0.093 | **0.042** | 0.073 | **0.046** | 0.088 | 0.075 | 0.090 |
|  |  | **Total** | 0.064 | 0.063 | **0.037** | 0.468 | 0.251 | 0.107 | 0.082 | 0.088 | 0.314 | **0.021** | **0.032** | 0.077 | **0.043** | 0.106 | **0.045** | 0.073 |
| **Meat consumption**   - **0-1 time days/week** - **2-4 times days/week** - **5-7 times days/week** | **16**  **125**  **3** | **Female** | **0.049** | **0.042** | 0.093 | 0.184 | 0.307 | 0.083 | **0.039** | 0.113 | 0.090 | **0.029** | **0.038** | 0.123 | 0.102 | **0.050** | 0.299 | 0.106 |
|  |  | **Male** | 0.432 | 0.286 | 0.455 | 0.434 | 0.416 | 0.446 | 0.482 | 0.418 | 0.309 | 0.492 | 0.346 | 0.369 | 0.315 | 0.445 | 0.340 | 0.373 |
|  |  | **Total** | 0.202 | 0.261 | 0.261 | 0.402 | 0.443 | 0.239 | 0.139 | 0.254 | 0.210 | 0.073 | 0.100 | 0.228 | 0.306 | 0.155 | 0.468 | 0.258 |
| **Face mask**   - **0-1 time days/week** - **2-4 times days/week** - **5-7 times days/week** | **110**  **32**  **2** | **Female** | 0.122 | 0.483 | 0.290 | 0.402 | 0.387 | 0.199 | 0.232 | 0.180 | 0.073 | 0.431 | 0.436 | 0.484 | 0.116 | 0.077 | 0.359 | 0.488 |
|  |  | **Male** | 0.428 | 0.346 | 0.179 | 0.238 | 0.328 | 0.411 | 0.460 | 0.335 | 0.474 | 0.058 | 0.078 | 0.370 | 0.367 | 0.432 | 0.378 | 0.473 |
|  |  | **Total** | 0.159 | 0.458 | 0.103 | 0.348 | 0.239 | 0.231 | 0.310 | 0.493 | 0.177 | 0.220 | 0.172 | 0.423 | 0.136 | 0.146 | 0.253 | 0.497 |
| **Insecticide exposure**   - **0-1 time days/week** - **2-4 times days/week** - **5-7 times days/week** | **109**  **35**  **0** | **Female** | **0.024** | **0.043** | **0.020** | 0.278 | 0.071 | 0.054 | **0.013** | 0.105 | 0.224 | 0.324 | 0.126 | **0.019** | **0.018** | 0.069 | 0.461 | **0.049** |
|  |  | **Male** | 0.492 | 0.488 | 0.221 | 0.108 | 0.439 | 0.477 | 0.498 | 0.286 | 0.182 | 0.242 | 0.324 | 0.492 | 0.438 | 0.286 | 0.153 | 0.465 |
|  |  | **Total** | 0.144 | 0.198 | 0.183 | 0.414 | 0.135 | 0.218 | 0.140 | 0.157 | 0.136 | 0.465 | 0.364 | 0.097 | 0.098 | 0.102 | 0.231 | 0.209 |
| **Smoking exposure**   - **0-1 time days/week** - **2-4 times days/week** - **5-7 times days/week** | **123**  **9**  **12** | **Female** | 0.317 | 0.212 | 0.497 | 0.292 | 0.225 | 0.379 | 0.346 | 0.259 | 0.189 | 0.154 | 0.209 | 0.286 | 0.326 | 0.485 | 0.295 | 0.167 |
|  |  | **Male** | 0.347 | 0.298 | 0.424 | 0.188 | 0.484 | 0.437 | 0.336 | 0.220 | 0.409 | 0.368 | **0.010** | 0.388 | 0.477 | 0.323 | 0.299 | 0.300 |
|  |  | **Total** | 0.232 | 0.164 | 0.428 | 0.298 | 0.268 | 0.358 | 0.276 | 0.180 | 0.123 | 0.206 | **0.026** | 0.300 | 0.335 | 0.352 | 0.300 | 0179 |
| **Incense exposure**   - **0-1 time days/week** - **2-4 times days/week** - **5-7 times days/week** | **37**  **74**  **33** | **Female** | **0.041** | 0.159 | **0.048** | 0.094 | **0.037** | **0.035** | 0.061 | 0.053 | 0.256 | 0.172 | 0.282 | 0.081 | **0.044** | 0.058 | 0.460 | 0.233 |
|  |  | **Male** | 0.323 | 0.115 | 0.211 | 0.140 | 0.331 | 0.206 | 0.218 | 0.429 | 0.496 | 0.407 | 0.353 | 0.164 | 0.391 | 0.168 | 0.289 | 0.282 |
|  |  | **Total** | 0.331 | 0.263 | 0.368 | **0.024** | 0.254 | 0.436 | 0.470 | 0.293 | 0.446 | 0.352 | 0.248 | 0.466 | 0.273 | 0.477 | 0.431 | 0.451 |
| **Fish consumption**   - **0-1 time days/week** - **2-4 times days/week** - **5-7 times days/week** | **57**  **87**  **0** | **Female** | 0.480 | 0.287 | 0.298 | 0.379 | 0.402 | 0.397 | 0.493 | 0.371 | 0.450 | 0.120 | 0.427 | 0.371 | 0.419 | 0.448 | 0.109 | 0.237 |
|  |  | **Male** | 0.162 | 0.145 | 0.149 | 0.306 | 0.194 | 0.169 | 0.125 | 0.241 | 0.497 | 0.079 | 0.165 | 0.086 | 0.184 | 0.173 | 0.251 | 0.193 |
|  |  | **Total** | 0.250 | 0.138 | 0.447 | 0.420 | 0.252 | 0.280 | 0.179 | 0.213 | 0.457 | 0.065 | 0.197 | 0.114 | 0.301 | 0.216 | 0.114 | 0.397 |
| **Omega 3 consumption**   - **0-1 time days/week** - **2-4 times days/week** - **5-7 times days/week** | **117**  **19**  **8** | **Female** | 0.172 | 0.153 | 0.139 | 0.229 | 0.068 | 0.224 | 0.227 | 0.425 | 0.082 | 0.091 | 0.229 | 0.216 | 0.125 | 0.168 | 0.222 | 0.170 |
|  |  | **Male** | 0.391 | 0189 | 0.403 | 0.362 | 0.458 | 0.402 | 0.403 | 0.308 | 0.409 | 0.496 | 0.126 | 0.442 | 0.385 | 0.353 | 0.197 | 0.291 |
|  |  | **Total** | 0.215 | 0.082 | 0.150 | 0.150 | 0.145 | 0.266 | 0.289 | 0.302 | 0.118 | 0.109 | 0.247 | 0.390 | 0.189 | 0.201 | 0.095 | 0.164 |
| **Dietary supplement intake**   - **0-1 time days/week** - **2-4 times days/week** - **5-7 times days/week** | **100**  **28**  **16** | **Female** | 0.380 | **0.046** | 0.288 | 0.499 | 0.073 | 0.401 | 0.495 | 0.380 | 0.452 | 0.194 | 0.054 | 0.394 | 0.452 | 0.471 | 0.256 | 0488 |
|  |  | **Male** | 0.172 | 0.429 | 0.159 | 0.129 | 0.167 | 0.287 | 0.184 | 0.207 | 0.068 | **0.048** | 0.275 | 0.084 | 0.316 | 0.285 | 0.125 | 0.251 |
|  |  | **Total** | 0.281 | 0.229 | 0.417 | 0.431 | 0.051 | 0.371 | 0.216 | 0.253 | 0.253 | 0.453 | 0.302 | 0.107 | 0.332 | 0.308 | 0.338 | 0.294 |
