# Supplementary material for: Cross-sectional study on urinary metal concentrations in young adult residents of Emirate of Sharjah, United Arab Emirates
Source: PLoS One. 2024 Nov 5;19(11):e0312964. doi: 10.1371/journal.pone.0312964 (PMC11537376; doi:10.1371/journal.pone.0312964)
Supplement: S2 Table — (DOCX) [file pone.0312964.s002.docx]

**Table S2: Independent-Samples Mann-Whitney U Test Summary.**

| **Independent-Samples Mann-Whitney U Test Summary** | | | | | | | | | | | | | | | | | |
| --- | --- | --- | --- | --- | --- | --- | --- | --- | --- | --- | --- | --- | --- | --- | --- | --- | --- |
|  | **Tl** | **Cd** | **Co** | **B** | **Mn** | **Cu** | **Ag** | **Mg** | **Sr** | **Ba** | **Cr** | **Fe** | **Ni** | **Al** | **Pb** | **As** |  |
| Mann-Whitney U | 2053.5 | 2089.5 | 2120 | 1929 | 2255 | 2053 | 2529.5 | 2188 | 2279 | 2650 | 2198.5 | 2450 | 1963 | 2166 | 2187 | 2138 |  |
| Wilcoxon W | 4609.5 | 4645.5 | 4676 | 4485 | 4811 | 4609 | 5085.5 | 4744 | 4835 | 5206 | 4754.5 | 5006 | 4519 | 4722 | 4743 | 4694 |  |
| Asymp. Sig. (2-tailed) | **0.032** | **0.045** | 0.060 | **0.008** | 0.179 | **0.031** | 0.804 | 0.107 | 0.212 | 0.815 | 0.116 | 0.572 | **0.012** | 0.089 | 0.106 | 0.070 |  |
