# Supplementary figures and images for: Cross-sectional study on urinary metal concentrations in young adult residents of Emirate of Sharjah, United Arab Emirates
Source: PLoS One. 2024 Nov 5;19(11):e0312964. doi: 10.1371/journal.pone.0312964 (PMC11537376; doi:10.1371/journal.pone.0312964)

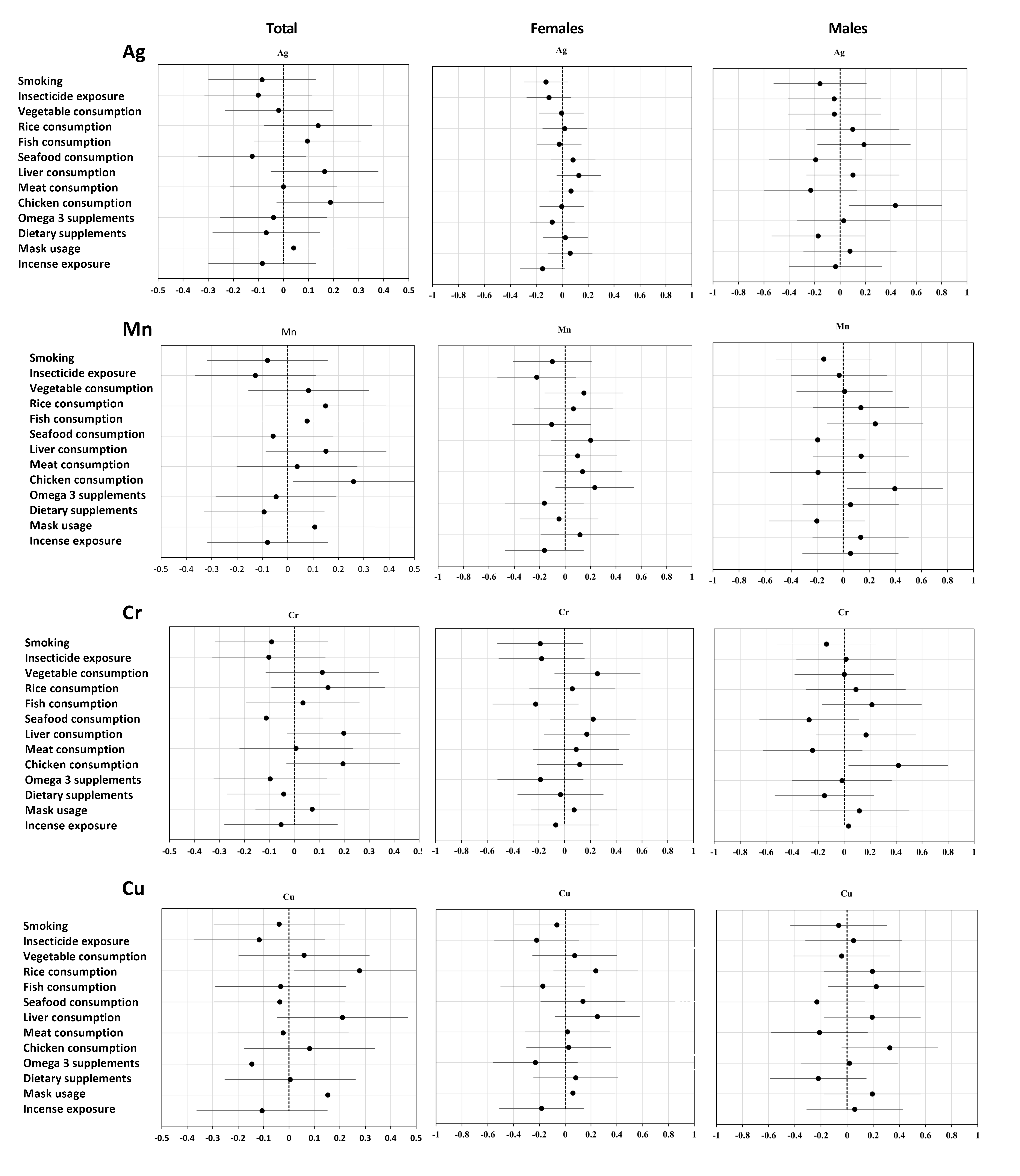

Supplement: S1 Fig — (TIF) [file pone.0312964.s003.tif]

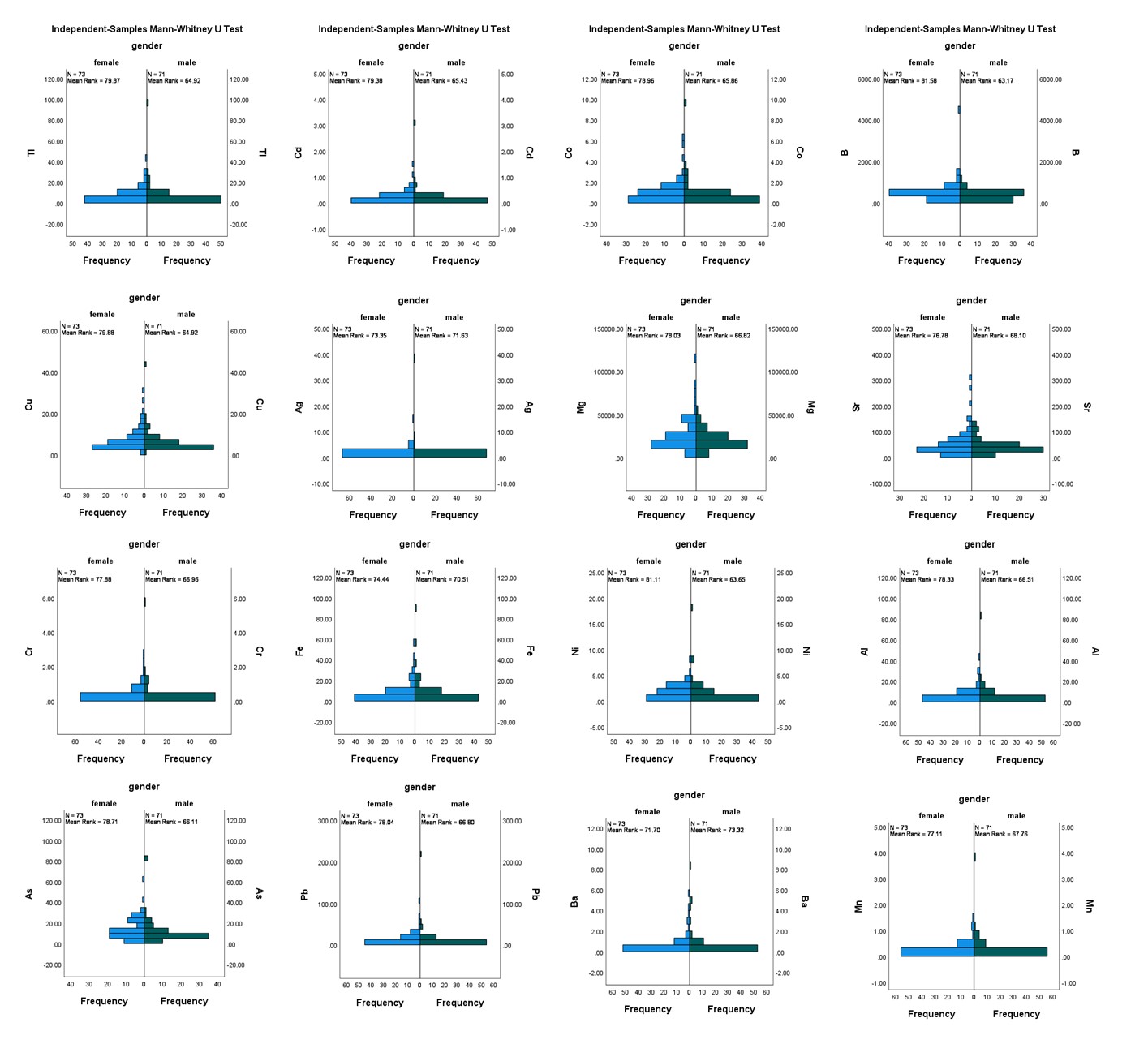

Supplement: S2 Fig — (JPG) [file pone.0312964.s004.jpg]
